# Effects of dietary selenium yeast supplementation on production performance, apparent nutrient digestibility, serum antioxidant parameters, and selenoprotein content in velvet antlers of sika deer (*Cervus nippon*)

**DOI:** 10.3389/fvets.2025.1735748

**Published:** 2026-01-09

**Authors:** Weili Sun, Cong Huang, Hongpeng Shi, Guangyu Li, Zhen Liu, Lisheng Zhou, Haiping Zhao

**Affiliations:** 1 College of Animal Science and Technology, Qingdao Agricultural University, Qingdao, China; 2 Shandong Engineering Research Center of Livestock Products Functional Utilization, Qingdao, China; 3 Institute of Special Animal and Plant Sciences, Chinese Academy of Agricultural Sciences, Changchun, China; 4 National Genetic Evaluation Centre of Special Livestock, Qingdao, China

**Keywords:** apparent nutrient digestibility, production performance, selenium yeast, selenoprotein, serum antioxidant parameters, sika deer

## Abstract

**Introduction:**

Selenium is an essential trace element for the growth of sika deer (*Cervus nippon*). However, the optimal dietary supplementation level and its effects on growth performance, nutrient metabolism, and antioxidant capacity in the deer, as well as selenoprotein synthesis in velvet antlers, remain unclear. This study aimed to investigate the effects of different dietary selenium yeast levels on these key physiological and molecular indicators.

**Methods:**

Twenty healthy, 5-year-old male sika deer at the antler growth stage were randomly assigned to four groups (*n* = 5): SY1 (control, 0 mg/kg Se), SY2 (0.3 mg/kg Se), SY3 (1.2 mg/kg Se), and SY4 (4.8 mg/kg Se). The trial included a 7-day adaptation period followed by a 60-day experimental period. Production performance, apparent nutrient digestibility, serum antioxidant status, and selenoprotein expression (GPX4 and SELENOP) in different antler tissue layers were systematically evaluated.

**Results:**

No significant differences were observed among groups in antler weight and main dimensional indices. Apparent digestibility of dry matter, calcium, and phosphorus in SY2 was significantly higher than in SY1 and SY3. Serum glutathione peroxidase (GSH-Px) and thioredoxin reductase (TrxR) activities in SY2 were significantly elevated compared with SY1 (*p* < 0.05). Selenoprotein analysis indicated that SELENOP protein expression in all antler tissue layers of SY3 was significantly higher than in other groups, whereas GPX4 protein expression was significantly upregulated in SY2.

**Conclusion:**

Dietary supplementation with 0.3 mg/kg selenium yeast effectively improved nutrient digestibility and serum antioxidant capacity in sika deer. Furthermore, both 0.3 mg/kg and 1.2 mg/kg selenium yeast enhanced the synthesis of key selenoproteins (GPX4 and SELENOP) in velvet antlers. These findings provide a scientific basis for selenium nutrition strategies during the antler growth stage and support the development of selenium-enriched velvet antler products.

## 1 Introduction

Sika deer (*Cervus nippon*), as a traditionally economically valuable medicinal species, produce antlers containing diverse bioactive compounds that exert antioxidant, antitumor, anti-osteoporotic, and wound-healing effects. These properties underpin their extensive utilization in pharmaceutical, nutraceutical, and dermocosmetic applications (1, 2). Antler initiation and regeneration are coregulated by sex hormones and insulin-like growth factor-1 (IGF-1) (3). Nevertheless, nutritional factors critically determine the temporal precision of antler growth initiation and cyclical regeneration programming (4). While selenium requirements for ruminants such as cattle and sheep have been established (5), optimal Se regimens for sika deer remain undefined due to their shorter domestication history. Given the distinct nutritional demands of antler-producing deer, elucidating the effects of Se on growth performance, nutrient metabolism, and antler quality carries substantial implications for production practices.

Selenium (Se), an essential trace element for animal growth, elicits immune dysregulation, growth retardation, and reproductive dysfunction when deficient (6, 7). In natural systems, Se exists predominantly in organic and inorganic forms, with organic Se demonstrating superior bioavailability and reduced toxicity compared to inorganic analogs (8). Dietary organic Se supplementation mitigates heavy metal accumulation in deer tissues while enhancing systemic antioxidant capacity (9).

Selenoproteins constitute a class of proteins incorporating selenocysteine (Sec) as the 21st amino acid (10). GPX4 is the only GPX enzyme capable of reducing large, structurally complex lipid hydroperoxides. It protects lipids and biomembranes from oxidative degradation and serves as a key regulator of ferroptosis (11). SELENOP, synthesized primarily in the liver, mediates systemic selenium distribution via the SELENOP recycling pathway while concurrently exerting antioxidant effects (12). Critically, sika deer during the velvet antler stage undergo sequential physiological challenges: Casting of antler pedicles, Rapid wound-healing, Accelerated antler regeneration (13). These processes generate substantial reactive oxygen species (ROS) due to high metabolic demands. Consequently, robust antioxidant and anti-inflammatory responses are indispensable for producing high-quality antlers (14).

This study aims to evaluate the effects of graded selenized yeast supplementation on production performance, nutrient apparent digestibility, serum antioxidant parameters, and antler selenoprotein content in sika deer, thereby determining the optimal dietary selenium level to establish a scientific basis for rational selenium utilization during the velvet antler stage and production of selenium-enriched premium antlers.

## 2 Materials and methods

### 2.1 Animals and experimental design

A total of 20 healthy antler-growing male sika deer aged 5 years with similar body condition were selected and randomly divided into four groups with five replicates per group and one deer per replicate. Each group was separately penned. Among them, the SY1 group served as the control group and was fed a basal diet without additional selenium supplementation. The SY2, SY3, and SY4 groups were experimental groups fed diets supplemented with selenium yeast (provided by Zhengzhou Huafeng Food Technology Co., Ltd., containing 2,300 mg/kg selenium) based on the basal diet, with selenium addition levels of 0.3, 1.2, and 4.8 mg/kg, respectively. Feeding amounts were determined according to the dietary requirements of sika deer during different periods. During the experiment, animals were fed twice daily at fixed times and had free access to water. All animal procedures were approved and authorized by the Animal Ethics Committee of Qingdao Agricultural University (Approval No. DKY20240423).

### 2.2 Diets

The basal diet was formulated according to the nutritional standards established by the NRC (15) and specific requirements for sika deer, combined with production practices. The composition and nutritional components of the basal diet are presented in Table 1. The nutritional content of the basal diet was consistent between the control and experimental groups. The control group received no additional selenium yeast supplementation, with tested dietary selenium content at 0.04 mg/kg. Based on the basal diet, the experimental groups received supplemental selenium at levels of 0.3, 1.2, and 4.8 mg/kg, respectively.

**Table 1:** The composition and nutrient levels of the basal diet (air-dry basis) %.

Items	Content
Ingredients	
Corn	42.57
Soybean meal	25.54
Maize germ	8.51
Wheat bran	5.96
Silage corn stalk	13.86
CaHPO_4_	1.28
NaHCO_3_	0.43
NaCl	0.85
Premix^a^	1.00
Total	100.00
Nutrient levels	
ME/(MJ/kg)	10.50
CP	19.07
EE	2.31
NDF	60.41
ADF	16.62
Ca	0.78
P	0.58
Se/(mg/kg)	0.04

a The premix provided the following per kg of diets: FeSO4·H2O 0.2 g, ZnSO4·H2O 0.15 g, MnSO4·H2O 0.06 g, CuSO4 0.025 g, VA 4000 IU, VD 1000 IU, Thr 0.6 g, Met 1 g, Lys 2 g, Peptide rhzomorph 1 g, Butyrin (50%) 1 g, Plant essential oil 0.5 g, Mildew free probiotics 0.7 g, Antioxygen 0.1 g.

### 2.3 Sample collection

On Days 1 and 60 before morning feeding, deer were anesthetized with xylazine hydrochloride for body weight measurement, and 10 mL of blood was collected from the jugular vein by certified technicians. Blood samples were centrifuged at 4,000 rpm for 10 min, and the serum was aliquoted into 1.5 mL centrifuge tubes and stored at −80 °C. From Day 30 onward, fecal samples were collected daily between 05:00 and 06:00 for three consecutive days for apparent digestibility analysis, with care taken to avoid contamination.

### 2.4 Growth performance

Body weights were recorded at the trial commencement and upon harvest of two-pronged antlers. Initial body weight (IBW) and final body weight (FBW) were measured following a 12-h overnight fast prior to morning feeding. Daily feed intake and orts were monitored for each deer throughout the trial period. Average daily gain (ADG), average daily feed intake (ADFI), dry matter intake (DMI), and feed-to-gain ratio (F/G) were calculated.

### 2.5 Antler production performance

Prior to antler resection, antler dimensions were measured using a flexible tape. Post-resection, antler weight was recorded. All data represent the average of left and right antlers. Pedicle distance (inter-pedicle span measured at skin level using calipers), pedicle circumference (mid-pedicle girth), main beam length (natural length from forklet base to apex measured posteriorly), main beam circumference (minimal girth at mid-beam), brow-tine length (natural length from forklet base to brow-tine tip), and brow-tine circumference (mid-tine girth) were quantified. Measurements were recorded to 0.1 cm precision.

### 2.6 Apparent nutrient digestibility

Dry matter (DM), crude protein (CP), ether extract (EE), acid detergent fiber (ADF), neutral detergent fiber (NDF), phosphorus (P), and calcium (Ca) the diets and feces were analyzed according to AOAC (16) method.

### 2.7 Determination of selenium content

Trace mineral concentrations in the diet, feces, and velvet antler tissues were analyzed using dissolved ash prepared according to the AOAC (16) method. Selenium content was subsequently determined by atomic fluorescence spectrometry (AFS-9130, Beijing Titan Instruments Co., Ltd., China).

### 2.8 Serum antioxidant parameters

Serum glutathione peroxidase (GSH-Px), superoxide dismutase (SOD), and catalase (CAT) activities, along with malondialdehyde (MDA) content and total antioxidant capacity (T-AOC), were determined using commercial assay kits (Biosino Biotechnology and Science, Inc., Beijing, China).

### 2.9 Reverse transcription quantitative PCR (RT-qPCR)

Total RNA was extracted from approximately 0.10 g of each antler tissue section using TRIzol reagent (Invitrogen, USA) following the manufacturer’s instructions. RNA concentration and purity were determined using a NanoDrop 2000 spectrophotometer (Thermo Fisher Scientific, USA). First-strand cDNA was synthesized from 1 μg of total RNA using the PrimeScript™ RT Reagent Kit (Takara, Japan) according to the manufacturer’s protocol. RT-qPCR was performed on an ABI 7500 Real-Time PCR System (Thermo Fisher Scientific, USA) using iTaq™ Universal SYBR® Green Supermix (Bio-Rad, USA). Gene-specific primers were designed with OligoPerfect™ Designer (Thermo Fisher Scientific) based on sequences obtained from the NCBI database; primer sequences are listed in Table 2. Glyceraldehyde-3-phosphate dehydrogenase (GAPDH) was used as the internal reference gene, and the relative mRNA expression levels of selenoproteins were calculated using the 2 − ΔΔCt method.

**Table 2:** Primer sequences used for RT-qPCR analysis.

Gene	Gene name	Template accession number	Oligonucleotide primer design (5′ to 3′ direction)	Amplicon length (bp)
GPX4	Glutathione Peroxidase 4	NM_174770.3	F: GATCAAAGAGTTCGCCGCTG R: CCATACCGCTTCACCACACA	198
SELENOP	Selenoprotein P	NM_174459.3	F: TCAGGTCTTCATCACCACCA R: CTTCAGCATCTTCACGGCTT	201
GAPDH	Glyceraldehyde-3-Phosphate Dehydrogenase	NM_001034034.2	F: ACATCAAGTGGGGTGATGCT R: GGCATTGCTGACAATCTTGA	201

### 2.10 Western blot analysis

Tissue samples were washed twice with phosphate-buffered saline (PBS) and lysed in RIPA buffer containing a protease inhibitor cocktail. Antler tissue layers were homogenized using a mechanical tissue disruptor, and the homogenates were centrifuged at 12,000 rpm for 15 min at 4 °C. The resulting supernatants were collected for protein quantification using the Bradford assay (17). Proteins were denatured at 95 °C for 5 min prior to electrophoresis. Western blot analysis was performed using the following antibodies: anti-GAPDH (AB181603), anti-GPX4 (AB231174), anti-SELENOP (AB193193), and goat anti-rabbit IgG secondary antibody (AB205718). Protein sequences and molecular weights were verified using UniProtKB, and all antibodies were purchased from Abcam (Cambridge, UK).

### 2.11 Statistical analysis

All statistical analyses were performed using SPSS 22.0. Data normality and homogeneity of variance were tested using the Shapiro–Wilk and Levene tests, respectively. One-way ANOVA was conducted to evaluate the effects of different dietary selenium yeast levels, followed by Duncan’s multiple range test for mean comparisons. Data are expressed as mean ± SD. Differences were considered significant at *p* < 0.05, and highly significant at *p* < 0.01.

## 3 Results

### 3.1 Growth performance

As shown in Table 3, dietary selenium supplementation levels had no significant effects (*p* > 0.05) on ADG and DMI of antler-growing sika deer. F/G in SY3 was lower than other groups, though not significantly (*p* > 0.05). Tissue selenium retention differed significantly (*p* < 0.01) among SY2, SY3, and SY4.

**Table 3:** Effects of dietary selenium yeast supplementation on growth performance in sika deer.

Items	Groups				*p*-value
	SY1	SY2	SY3	SY4	
Initial body weight/kg	98.25 ± 5.08	98.17 ± 6.20	98.50 ± 4.95	97.33 ± 2.72	0.995 1
Final body weight/kg	107.86 ± 6.65	103.31 ± 7.08	104.87 ± 4.18	104.67 ± 3.27	0.992 8
Average daily gain/(kg/d)	0.20 ± 0.06	0.19 ± 0.09	0.20 ± 0.07	0.21 ± 0.05	0.990 8
Dry matter intake/(kg/d)	2.10 ± 0.09	2.08 ± 0.08	2.07 ± 0.09	2.09 ± 0.08	0.993 9
Feed/gain	9.86 ± 0.45	12.49 ± 1.83	9.63 ± 1.89	13.36 ± 2.08	0.138 5
Selenium deposition in body/(mg/d)	—	0.66 ± 0.03^Cc^	1.39 ± 0.15^Bb^	4.83 ± 0.83^Aa^	<0.000 1

Values with no letters or the same letters are not significantly different (*p* > 0.05). Different lowercase superscripts indicate significant differences (*p* < 0.05), and different uppercase superscripts indicate highly significant differences (*p* < 0.01). The same applies below.

### 3.2 Antler production performance

As presented in Table 4, dietary selenium supplementation had no significant effect on antler weight in sika deer (*p* > 0.05). The mean antler weight in SY3 was numerically higher than in SY1, SY2, and SY4; however, these differences were not statistically significant (*p* > 0.05). All antler dimensional parameters in SY3 showed similar non-significant trends compared with the other groups (*p* > 0.05).

**Table 4:** Effects of dietary selenium yeast supplementation on velvet antler production performance in sika deer.

Items	Groups				*p*-value
	SY1	SY2	SY3	SY4	
Velvet antler weight/kg	1.39 ± 0.35	1.39 ± 0.40	1.63 ± 0.30	1.26 ± 0.36	0.603 4
Average daily gain of velvet antler/g	28.47 ± 6.56	27.23 ± 7.78	29.86 ± 6.75	23.07 ± 8.23	0.635 6
Velvet antler measurement indexes/cm					
Main beam length	26.28 ± 2.76	27.48 ± 3.93	27.76 ± 1.75	25.81 ± 3.14	0.792 0
Main beam circumference	15.75 ± 1.39	15.64 ± 1.70	17.05 ± 1.84	15.25 ± 1.54	0.518 3
Crown length	18.83 ± 2.66	19.59 ± 3.39	20.41 ± 1.56	17.90 ± 2.08	0.606 8
Crown circumference	15.49 ± 1.80	16.62 ± 1.66	18.40 ± 2.47	16.35 ± 2.22	0.332 7
Brow tine length	11.83 ± 1.02	11.24 ± 1.27	12.50 ± 1.55	10.31 ± 1.22	0.164 4
Brow tine circumference	9.01 ± 0.41	8.61 ± 0.61	9.24 ± 1.61	8.54 ± 0.47	0.672 7
Pedicle circumference	18.00 ± 1.15	18.05 ± 2.27	19.31 ± 0.65	17.71 ± 2.11	0.640 4
Pedicle distance	10.20 ± 1.72	11.50 ± 1.95	11.50 ± 1.06	11.10 ± 1.11	0.617 6

### 3.3 Apparent nutrient digestibility

As presented in Table 5, the apparent digestibility of DM, Ca, and P in group SY2 was significantly greater than SY1 and SY3 (*p* < 0.01). Concurrently, DM and P digestibility in SY2 exceeded SY4 (*p* < 0.05). For CP, no significant difference was observed between SY2 and SY1 (*p* > 0.05), whereas SY2 substantially surpassed SY3 (*p* < 0.01) and SY4 (*p* < 0.05). Selenium digestibility in SY2 was higher than SY3 and SY4 (*p* < 0.01).

**Table 5:** Effects of dietary selenium yeast supplementation on apparent nutrient digestibility in sika deer %.

Items	Groups				*p*-value
	SY1	SY2	SY3	SY4	
DM	66.02 ± 0.81^BCc^	72.44 ± 1.74^Aa^	65.60 ± 1.47^Bc^	69.20 ± 1.33^ACb^	<0.000 1
CP	74.48 ± 1.27^Aac^	78.86 ± 3.28^Aa^	59.47 ± 2.60^Bb^	66.74 ± 9.12^ABbc^	0.000 3
EE	82.74 ± 2.58	87.54 ± 2.73	83.73 ± 2.67	87.28 ± 2.77	0.046 7
NDF	75.66 ± 0.61^Bb^	79.42 ± 1.15^Aa^	73.98 ± 1.51^Bb^	75.84 ± 1.41^Bb^	<0.0001
ADF	49.09 ± 1.37^Bb^	55.78 ± 1.84^Aa^	46.70 ± 1.23^Bb^	48.20 ± 1.01^Bb^	<0.0001
Ca	45.75 ± 4.84^Bb^	59.85 ± 5.82^Aa^	44.78 ± 5.54^Bb^	51.60 ± 3.97^ABab^	0.002 5
P	24.79 ± 6.60^Bc^	55.25 ± 5.59^Aa^	26.94 ± 3.71^Bc^	42.83 ± 6.49^Ab^	<0.000 1
Se	—	89.01 ± 1.6^Aa^	48.87 ± 5.32^Bb^	42.48 ± 2.95^Bb^	<0.000 1

Values with no letters or the same letters are not significantly different (*p* > 0.05). Different lowercase superscripts indicate significant differences (*p* < 0.05), and different uppercase superscripts indicate highly significant differences (p < 0.01).

### 3.4 Serum antioxidant parameters

As shown in Table 6, serum CAT activity in SY4 was significantly greater than the control group (*p* < 0.05). Serum TrxR in SY2 was significantly higher than the control group (*p* < 0.05).

**Table 6:** Effects of dietary selenium yeast levels on serum antioxidant parameters in sika deer.

Item	Groups				*p-*value
	SY1	SY2	SY3	SY4	
GSH-Px	178.55 ± 18.15	190.05 ± 7.27	153.41 ± 5.42	162.86 ± 17.64	0.0458
SOD	10.32 ± 1.24	9.93 ± 1.07	10.48 ± 0.89	10.49 ± 0.65	0.8500
T-AOC	1.51 ± 0.04	1.52 ± 0.03	1.50 ± 0.04	1.48 ± 0.03	0.4764
CAT	1.63 ± 0.22^Ab^	1.81 ± 0.16^Aab^	1.96 ± 0.25^Aab^	2.92 ± 0.20^Aa^	0.0138
TrxR	3.07 ± 0.66^Ab^	5.14 ± 1.35^Aa^	3.26 ± 1.33^Aab^	3.25 ± 0.27^Aab^	0.0340
MDA	2.59 ± 0.25	2.58 ± 0.36	3.33 ± 0.30	3.46 ± 0.78	0.0360
NO	0.54 ± 0.22	0.60 ± 0.49	0.49 ± 0.23	0.60 ± 0.39	0.9695

Values with no letters or the same letters are not significantly different (*p* > 0.05). Different lowercase superscripts indicate significant differences (*p* < 0.05), and different uppercase superscripts indicate highly significant differences (*p* < 0.01).

### 3.5 Selenium content in antler tissue layers

**Figure 1:**
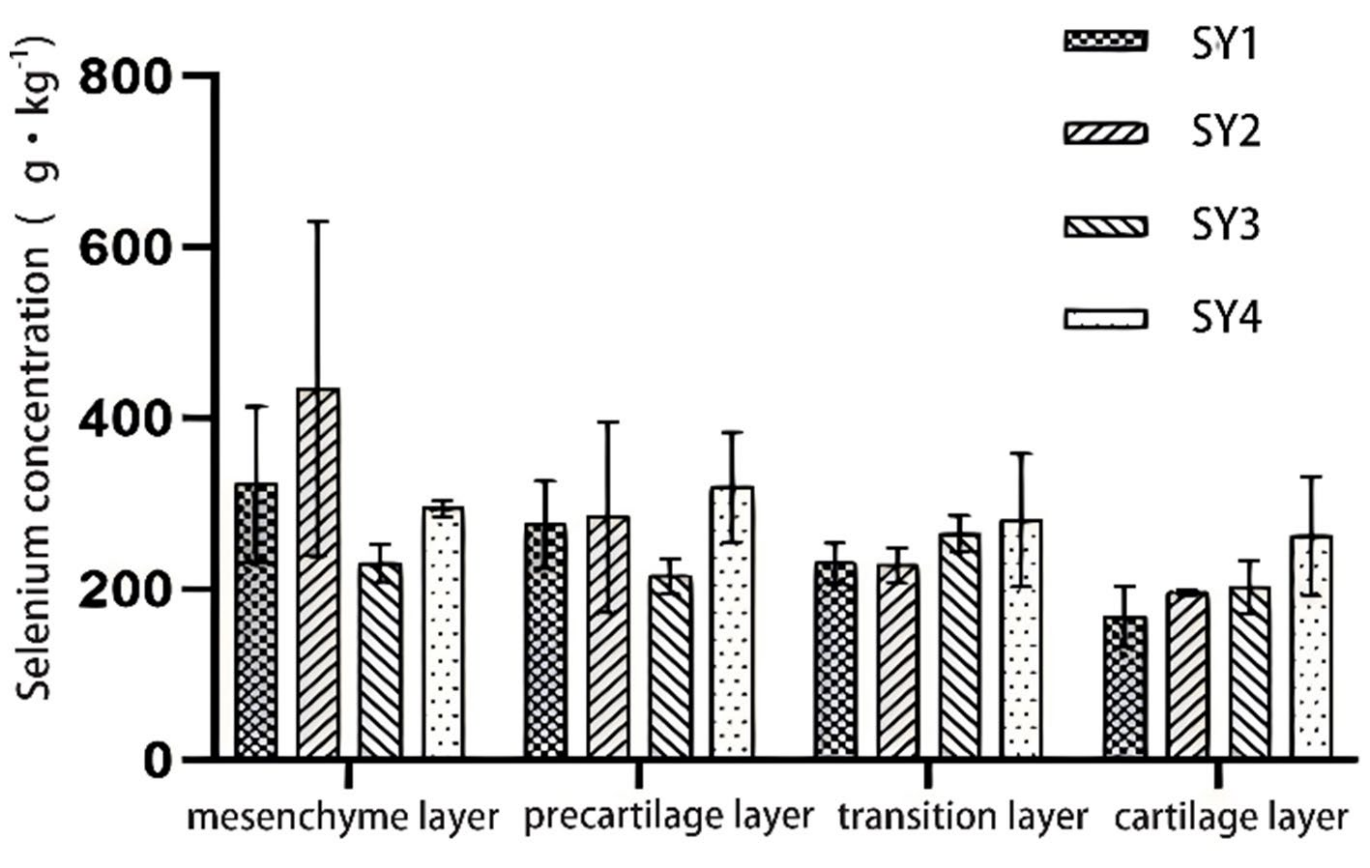
Selenium content in different tissue layers of velvet antler. * *p* < 0.05** *p* < 0.01. The same applies below.

As shown in Figure 1, yeast selenium had no significant effect on selenium content in different tissue layers of antler during the antler growth period of sika deer (*p* > 0.05).

### 3.6 Selenoprotein expression

**Figure 2:**
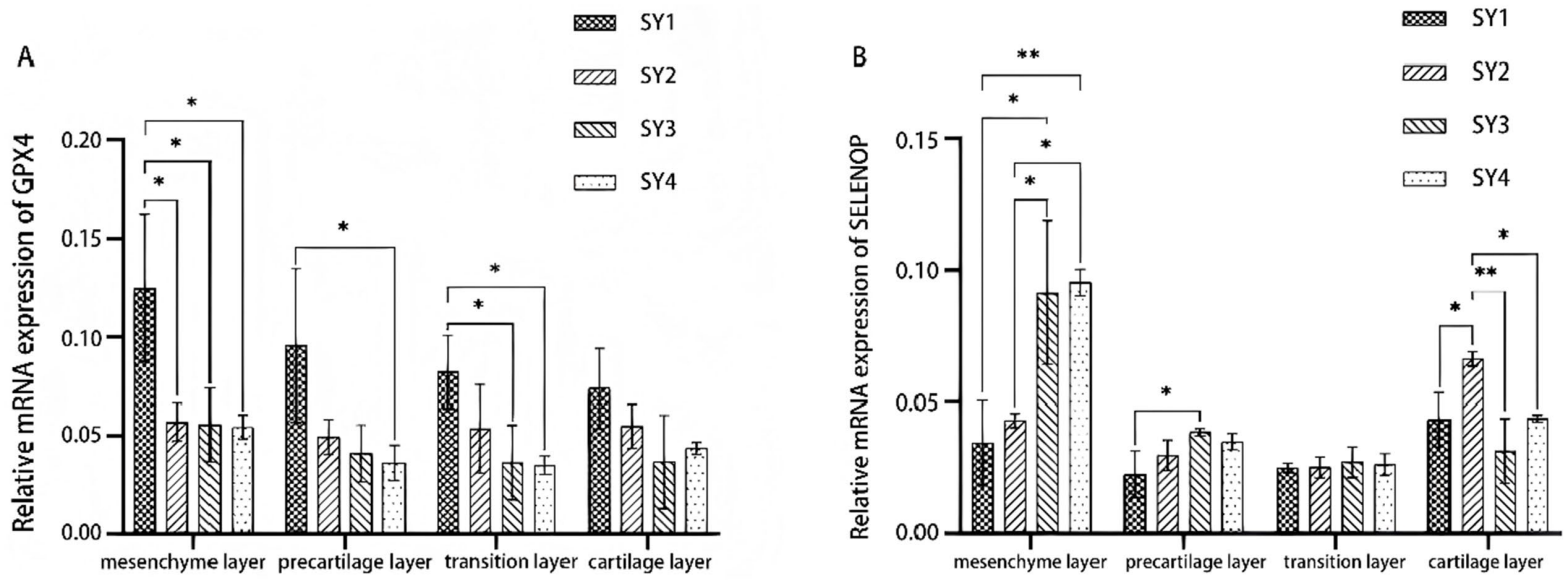
Relative expression levels of selenoprotein genes in different tissue layers of velvet antler. **(A)** Relative mRNA expression of GPX4 in mesenchymal, precartilage, transition, and cartilage layers. **(B)** Relative mRNA expression of SELENOP in the same layers.

As shown in Figure 2, the mRNA expression of GPX4 in the mesenchymal layer of sika deer velvet antlers was significantly higher in the SY1 group than in SY2, SY3, and SY4 (*p* < 0.05). In the precartilage and transition layers, GPX4 mRNA expression was significantly higher in SY1 than in SY4 (*p* < 0.05). The SELENOP mRNA expression in SY3 was significantly higher than in SY1 in both the mesenchymal and precartilage layers, whereas the highest SELENOP mRNA expression was observed in SY2 within the cartilage layer.

**Figure 3:**
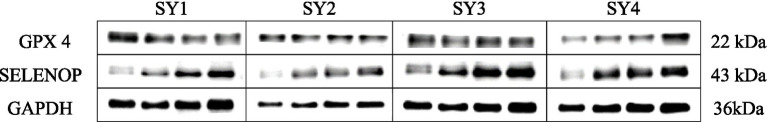
Western blot verification of selenoprotein in different tissue layers of velvet antler.

**Figure 4:**
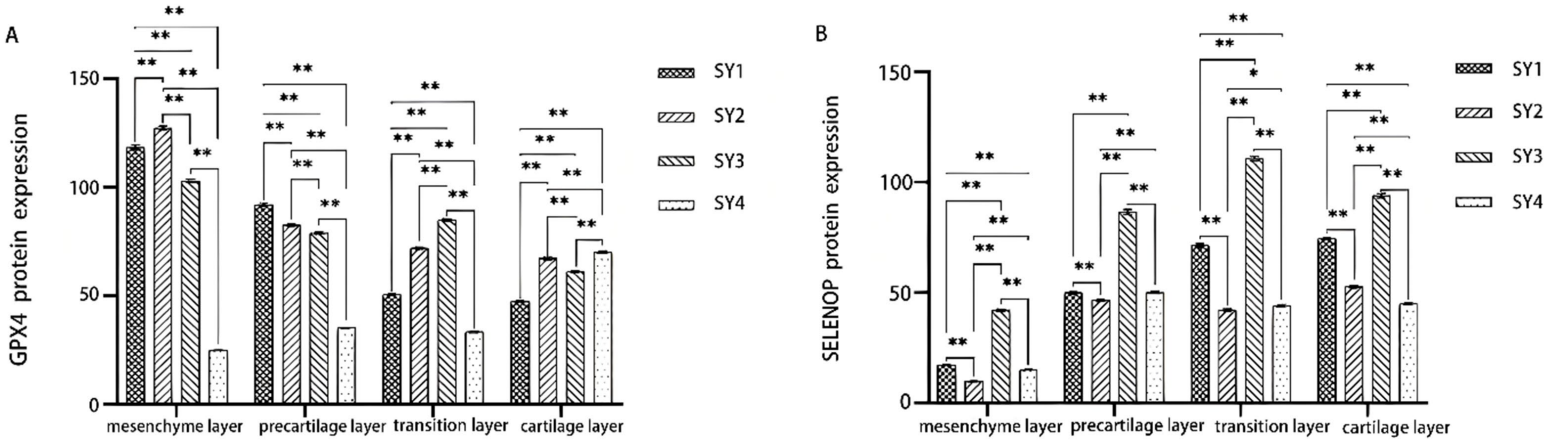
Relative expression levels of selenoprotein in different tissue layers of velvet antler. **(A)** GPX4 protein expression in mesenchymal, precartilage, transition, and cartilage layers. **(B)** SELENOP protein expression in the same layers.

As shown in Figures 3, 4, SY2 exhibited significantly higher GPX4 protein expression than SY1 in all tissue layers except the precartilage layer (*p* < 0.01), while its expression in the mesenchymal and precartilage layers was significantly greater than that in SY3 and SY4. For SELENOP, SY3 showed the highest overall SELENOP expression across the antler tissue layers (*p* < 0.01).

## 4 Discussion

Organic selenium exhibits higher bioavailability than inorganic forms in animals (8), enabling more efficient deposition in maternal tissues and subsequent transfer to offspring (18). In this trial, dietary supplementation with selenized yeast at 1.2 mg/kg selenium reduced the FGR in sika deer during the velvet antler stage, while ADG exhibited a quadratic response with increasing selenium levels. Previous studies indicate that selenium enhances ADG and feed efficiency in female sika deer (19). Jia et al. (20) observed no significant effects on ADG or ADFI in Tan sheep supplemented with selenized yeast. Consistent findings were reported by Silva et al. (21) in cattle and Liu et al. (22) in broilers. In the present study, ADG and ADFI similarly exhibited no significant changes in sika deer during the velvet antler stage. Consistent with these findings, ADG and ADFI in the present study remained statistically unchanged, potentially attributable to the high heritability of carcass traits, which may diminish responsiveness to nutritional interventions (23).

Selenium modulates antler growth, with serum selenium concentrations in wapiti during the velvet antler stage exceeding those in other physiological phases (24). Selenium enrichment occurs specifically at the antler apex (25), which serves as the primary growth center for antler development the primary growth center for antlerogenesis (3, 26). Selenoproteins potentially regulate both testosterone biosynthesis and insulin-like growth factor-1 (IGF-1) secretion (27). These mediators act synergistically within the periosteum to promote bone growth, repair, and metabolic homeostasis (28). This study observed that antler weight in sika deer supplemented with 1.2 mg/kg selenium increased by 0.24 kg compared to Group SY1 and SY2, and by 0.37 kg relative to Group SY4. This aligns with reported selenium-induced productivity enhancements in dairy cattle (29), likely attributable to selenium’s attenuation of oxidative stress, thereby improving physiological performance (30). Although the SY3 group showed numerically higher antler weight and dimensions, these differences were not statistically significant. Similar patterns have been reported in sika deer nutritional studies that measure velvet production and physiological responses (31). Moreover, antler growth is strongly influenced by tissue-specific developmental programs and endocrine status (32), which can mask treatment effects in studies with limited sample sizes. Therefore, the higher values observed in SY3 should be interpreted as an observational trend rather than proof of a biological effect, and require validation in larger trials.

Antler growth mobilizes substantial nutrients and mineral elements from systemic reserves to support rapid tissue development (33, 34). Bao et al. (19) demonstrated that dietary selenium supplementation at 0.3 mg/kg significantly improved apparent digestibility of CP and EE in sika deer. Similarly, selenohydroxy-methionine enhances apparent digestibility of CP, NDF, ADF, and selenium in lactating dairy cows while promoting rumen fermentation (35). This study observed that dietary selenium supplementation at 0.3 mg/kg improved the apparent digestibility of DM, CP, CF, Ca, P, and Se in sika deer to varying extents. This phenomenon may be attributed to selenium-induced modulation of gastrointestinal enzyme activity and rumen microbiota structure, thereby converting refractory nutrients into absorbable forms (36). Critically, selenium participates in GPX biosynthesis, protecting digestive organs against ROS and free radicals. This protective effect potentiates *α*-amylase and protease activity, ultimately enhancing protein and lipid digestion, absorption, and utilization (37).

This study found that graded selenized yeast supplementation exerted no significant effects on selenium content across distinct antler tissue layers in sika deer, while significantly modulating serum antioxidant status. This phenomenon may stem from selenium redistribution within the organism, where the element is transported to sites of oxidative stress to maintain systemic selenium homeostasis (38). GSH-Px reduces intracellular hydrogen peroxide levels, protecting lipids, proteins, and DNA from oxidative damage, whereas TrxR balances cellular redox status by generating reduced thioredoxin (11). Selenium supplementation in wapiti markedly elevates serum and hepatic selenium concentrations (39). Mousaie et al. (40) demonstrated that dietary selenized yeast enhances serum T-AOC alongside GSH-Px and SOD activities, while reducing MDA levels in lambs. Similarly, Gong et al. (41) observed increased serum GSH-Px, SOD, and CAT activities with improved T-AOC in dairy cows supplemented with selenized yeast at 7 and 12 days postpartum. In this trial, supplementation with 0.3 mg/kg selenium significantly enhanced serum TrxR activity and exhibited a tendency to elevate GPX and CAT activities. This likely occurs through selenium incorporation into the active sites of GSH-Px and TrxR, thereby activating antioxidant enzymes to mitigate ROS-induced damage (42). The limited response observed in the SY4 group (4.8 mg/kg) may reflect a biological plateau in selenium utilization, where increasing dietary selenium beyond the requirement does not further enhance production performance, antioxidant responses, or selenoprotein synthesis. Similar diminishing-return patterns have been reported in ruminants when selenium intake surpasses functional needs (43). Another possible explanation is that excessive selenium may exert pro-oxidant effects, even when supplied as organic selenium yeast. Studies in cattle and goats have shown that high selenium intake can increase oxidative stress markers or reduce antioxidant enzyme efficiency, suggesting a shift from antioxidant to pro-oxidant activity under excessive supplementation (44). Therefore, the attenuated response in SY4 may result from either a plateau effect or early pro-oxidant pressure, which could partially counteract the expected benefits.

Currently, 25 selenoproteins have been identified in mammals, and in our preliminary (unpublished) study, 13 selenoproteins were detected in velvet antlers, including members of the glutathione peroxidase (GPX) family, thioredoxin reductase (TXNRD) family, and selenoprotein P (SELENOP). In this study, two selenoproteins with multiple functional activities, GPX4 and SELENOP, were selected to investigate the effects of dietary selenium supplementation on their content in velvet antlers. In this study, dietary selenium supplementation downregulated GPX4 gene expression while enhancing selenoprotein abundance in the mesenchymal, transitional, and cartilaginous layers of velvet antlers. The abundance of GPX4 protein is transcriptionally regulated by the nuclear factor erythroid 2-related factor 2 (Nrf2) pathway (45). Previous studies have demonstrated that GPX4 undergoes multiple post-translational modifications (PTMs) that affect its stability and catalytic activity, suggesting that organic selenium may improve GPX4 stability and antioxidant function by modulating PTM processes (46). During wound healing, selenoproteins such as GPX4 and SELENOP play pivotal roles in the inflammatory phase by dampening pro-inflammatory cytokine responses and neutralizing peroxynitrite radicals, thereby mitigating oxidative stress and promoting tissue repair (38). Proteomic profiling of velvet antlers during the growth stage has revealed significant enrichment in lipid metabolism, superoxide radical elimination, and glutathione metabolism at the antler tip (47). During the rapid growth phase, elevated cellular metabolism triggers bursts of reactive oxygen species (ROS), and GPX4 plays a critical role in protecting antler cells from oxidative and free radical damage (48). In parallel, selenium supplementation significantly increased SELENOP gene and protein expression in velvet antlers. This may be attributed to the function of SELENOP as a selenium transport protein, facilitating selenium delivery and promoting selenoprotein synthesis when selenium availability is elevated. Functionally, SELENOP catalyzes phospholipid hydroperoxide degradation through its intrinsic glutathione peroxidase activity, thereby maintaining membrane stability. After hepatic uptake, selenium is incorporated into SELENOP for systemic distribution to peripheral tissues (49). Critically, selenium supplementation promoted antler selenoprotein synthesis, optimizing bioactive components through enhanced antioxidant capacity, thereby providing a theoretical foundation for developing value-added antler products with fortified health benefits.

## 5 Conclusion

In conclusion, dietary supplementation with 0.3 mg/kg selenium yeast effectively enhanced nutrient digestibility and serum antioxidant capacity in sika deer. Notably, both 0.3 and 1.2 mg/kg selenium yeast promoted selenoprotein synthesis in velvet antlers, resulting in selenium-enriched high-quality antlers.

## Data Availability

The raw data supporting the conclusions of this article will be made available by the authors, without undue reservation.
